# A contaminant-free assessment of Endogenous Retroviral RNA in human plasma

**DOI:** 10.1038/srep33598

**Published:** 2016-09-19

**Authors:** Timokratis Karamitros, Dimitrios Paraskevis, Angelos Hatzakis, Mina Psichogiou, Ioannis Elefsiniotis, Tara Hurst, Anna-Maria Geretti, Apostolos Beloukas, John Frater, Paul Klenerman, Aris Katzourakis, Gkikas Magiorkinis

**Affiliations:** 1 Department of Zoology, University of Oxford, Oxford, United Kingdom; 2 Department of Hygiene, Epidemiology and Medical Statistics, Medical School, University of Athens, Athens, Greece; 3 First Department of Internal Medicine, Medical School, University of Athens, Athens, Greece; 4 Academic Department of Internal Medicine-Hepatogastroenterology, General and Oncology Hospital ‘Agioi Anargyroi’, Athens, Greece; 5 Institute of Infection and Global Health, University of Liverpool, Liverpool, United Kingdom; 6 Nuffield Department of Medicine, University of Oxford, Oxford, United Kingdom.

## Abstract

Endogenous retroviruses (ERVs) comprise 6–8% of the human genome. HERVs are silenced in most normal tissues, up-regulated in stem cells and in placenta but also in cancer and HIV-1 infection. Crucially, there are conflicting reports on detecting HERV RNA in non-cellular clinical samples such as plasma that suggest the study of HERV RNA can be daunting. Indeed, we find that the use of real-time PCR in a quality assured clinical laboratory setting can be sensitive to low-level proviral contamination. We developed a mathematical model for low-level contamination that allowed us to design a laboratory protocol and standard operating procedures for robust measurement of HERV RNA. We focus on one family, HERV-K HML-2 (HK2) that has been most recently active even though they invaded our ancestral genomes almost 30 millions ago. We extensively validated our experimental design on a model cell culture system showing high sensitivity and specificity, totally eliminating the proviral contamination. We then tested 236 plasma samples from patients infected with HIV-1, HCV or HBV and found them to be negative. The study of HERV RNA for human translational studies should be performed with extensively validated protocols and standard operating procedures to control the widespread low-level human DNA contamination.

Endogenous retroviruses (ERVs) are the result of ancient retroviral infections that have integrated in the germline of the host^1^ and represent a >100-million-years “fossil record” of such retroviral infections. Human ERVs (HERVs) are present in more than four hundred thousand copies and comprise almost 8% of the human genome^1^,^2^. They are classified in several families, each family representing an independent retroviral invasion and colonization of the germline.

HERV-K HML-2 (HK2) entered our ancestors’ genomes around 30 million years ago, but continued to integrate even after the human-chimpanzee divergence^3^. HERVs that invaded the germline more recently (i.e. during the last 5 million years) are likely to be insertionally polymorphic among individuals, but also likely not to have accumulated mutations or recombination events that would render them non-functional^4^. Thus, functional open reading frames for the *gag*, *pol* or *env* genes exist in some HK2 proviruses and can be expressed to produce virus-like particles^5^,^6^,^7^,^8^. Regardless of their coding capacity, HERVs are suppressed mostly through epigenetic modifications like CpG methylation^9^,^10^. Consistent hypomethylation of human transposons has been shown in malignancies^11^,^12^, suggesting that this might be an important mechanism for the up-regulation of HERVs during a wide spectrum of cancers^13^,^14^,^15^,^16^.

HERVs are also suppressed by intrinsic restriction factors that act on other retroviruses^17^,^18^; these include the enzyme TREX1, the loss of which results in the accumulation of endogenous retrovirus DNA and is associated with the disease Aicardi-Goutières Syndrome (AGS)^19^. Thus co-infections with pathogens that antagonize these restriction factors could allow the “opportunistic expression” of HERVs. Indeed, elevated expression of HERVs has been reported during other viral infections e.g. from human cytomegalovirus^20^ and human herpesvirus^21^. Since HK2 integrations, which can produce all the protein products, are present in the genome, it is feasible that opportunistic up-regulation could result in the production of HK2 viral particles. Although no HK2 provirus has been shown to be infectious up to now, there still exists the possibility that a polymorphic infectious provirus would be circulating^22^, but also the “opportunistic up-regulation” could in theory result in producing HK2 infectious virions through recombination^23^,^24^,^25^.

HERV expression can be used for the development of clinical applications, for example as a biomarker of unknown viral co-infections, sub-clinical cancer or autoimmune disorders. On the other hand the research of HERVs on translational applications has faced a history of conflicting results. For example, HK2 has reportedly been detected in the plasma of HIV-1 infected individuals, as well as lymphoma and breast cancer patients and the titers of HK2 viremia were associated with the treatment and outcome of these diseases^26^,^27^,^28^. Another group has shown that HIV-1 infection leads to increased transcription of HK2 proviruses, but did not verify the circulation of HK2 RNA in the plasma of HIV-1 infected patients^29^. Other observations include the presence of HK2-specific immune responses in the plasma of HIV-1-infected individuals^27^,^30^,^31^.

Here we describe the development of an approach comprises the laboratory setting, standard operating procedures, a highly efficient DNA decontamination and a highly sensitive molecular beacon quantitative PCR assay for the detection of HERV RNA in clinical samples. Although we focus on HK2, which is the most recently active, and thus is on the “spotlight” of translational studies, we suggest that similar approaches are essential for any translational study of HERV expression and human disease.

## Results

### Range and specificity of molecular beacon

**Figure 1:**
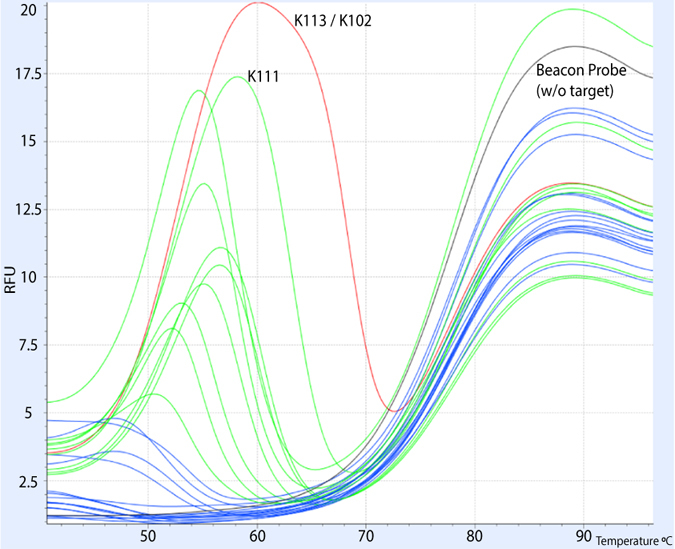
Multiple melt curves of HK2 *pol* molecular beacon probe against all possible proviral sequences. Beacon alone in black, correct target (K113) in red, detectable targets in green, undetectable targets in blue.

Detecting nucleic acids from a specific family of HERVs faces two major challenges: 1) the multiple integrations of the family in the genome might carry random/deleterious mutations and, 2) similar sequences from unrelated HERV families are likely to exist. These issues can lead to false negative (failure to detect mutated proviruses) or false positive (detection of proviruses from other families) results respectively. We thus chose to use a molecular beacon-based assay, which is expected to have higher target specificity compared to standard SYBR green reactions, high enough to be able to detect and genotype SNPs^32^. Undoubtedly, this may come at the cost of failure to detect mutants with mismatches on the binding site of the beacon (narrow range of targets). To test the ability of our beacon to sufficiently detect the known HK2 proviruses, we performed a multiple melt curve analysis (Fig. 1) against synthesized targets of all of the available full-length HK2 *pol* sequences. Our design is able to capture 42 out of 93 HK2 proviruses^6^, including the K102 (1q22) and K111 loci that were previously suggested to be upregulated in HIV-1 patients^33^,^34^. Out of the 51 undetectable proviruses (Table 1), 29 of them have extended deletions in the corresponding *pol* sequence target site, leaving 22 undetectable proviruses that are capable of encoding *pol*. Our assay has a narrower target range than the one described in Bhardwaj *et al.*^29^, however covers all the HK2 integrations that have been previously reported to be activated as well as the majority of the *pol* encoding proviruses, which can be replication competent and clinically relevant in a variety of diseases.

### DNase treatment for the high DNA:RNA ratio of non-cellular clinical samples

A standard DNase treatment can efficiently remove genomic DNA (gDNA) in the case of single-copy (or low-copy number) genes when the expected ratio of RNA:DNA is larger than (or even close to) one. In the case of HK2, the full-genome proviral copies are in the order of 100–200 per diploid cell. Assuming that 1 ml of plasma would have circulating gDNA equivalent to 100 cells, then a low HK2 viremia (i.e. 100 RNA copies per ml) with a standard nucleic acid extraction from the plasma would result into a ratio of RNA:DNA of 1:100 (or even lower). Indeed, our preliminary analyses where we ran qPCR in plasma RNA extractions without DNAse treatment indicated that the free HK2 DNA in nucleic acid eluates from human plasma extraction is in the order of 10^4^–10^5^ copies per ml. The detection of low-copy RNA in the background of high gDNA contamination requires very efficient DNA removal while, at the same time, preserving the sensitive RNA from experimental manipulation. Thus, we optimized the DNase treatment (see below), assessing extensively the removal of proviral gDNA and validating that RNA does not disintegrate. To make sure that the removal of gDNA is total and robust throughout the experiments, for each sample we ran mock-RT (−) (no RT enzyme added) qPCR reaction within the same experiment.

We tested the efficiency and the effects of turbo DNase inactivation after use of the proprietary inactivating reagent, or applying heat-based inactivation or adjusting a reagent followed-by-heat inactivation. We found that after using the dedicated inactivation reagent provided in the turbo DNAse kit, we were losing sensitivity in the low-copy (10^3^–10^2^ copies per reaction) range of the RNA standards, suggesting that either remnant DNase activity or PCR inhibition was present. We also observed loss of low-copy RNA standard sensitivity when we heat-inactivated the DNase, likely due to the disintegration of RNA in high temperature in the presence of divalent cations. Thus, we concluded that it was critical to add the inactivating reagent followed by a heating inactivation step because, apart from sequestering DNase, the reagent removes divalent cations that were present in the DNase digestion buffer. The use of inactivating reagent followed by heat inactivation restores the sensitivity in the low-copy RNA standards.

To further assess that these DNase-treated samples do not have any remnant DNase activity or inhibit PCR, we spiked treated samples or pure water controls with 10^3^ and 10^2^ of DNA standards and compared their Ct values. We also performed a double DNase treatment on the low-copy RNA standards and we find no evidence of loss of RNA copies, as the Ct values in successive treatments did not change.

### Low-level contamination of HERVs in a clinical virus reference laboratory

To assess the environmental contaminant scenario, we ran pilot experiments in conditions of a reference virology lab setting. The lab operates a Quality Assurance System according to the requirements of the internationally recognized standard ISO 9001:2008. The lab space was occupied by up to 5 validated workers and the pre-PCR steps were performed in standard PCR hoods, where only pre-PCR steps were allowed (no HEPA filtration). The background routine work of the lab included HIV, HBV and HCV molecular diagnostics that never experienced issues of contamination for these pathogens. During these pilot experiments, we occasionally detected low-level human gDNA contamination (at the level of 10–100 copies/reaction) in the no-RT control reactions. The contamination had a random pattern; it was not always detectable within the same/corresponding RT and no-RT reactions, suggesting that it was not due to inefficient DNAse treatment, it was low-level and probably occurred during the preparation of the reactions. For example, there were cases where we observed a positive no-RT with negative RT qPCR reaction.

### Modeling and controlling environmental-genomic DNA contamination

**Figure 2:**
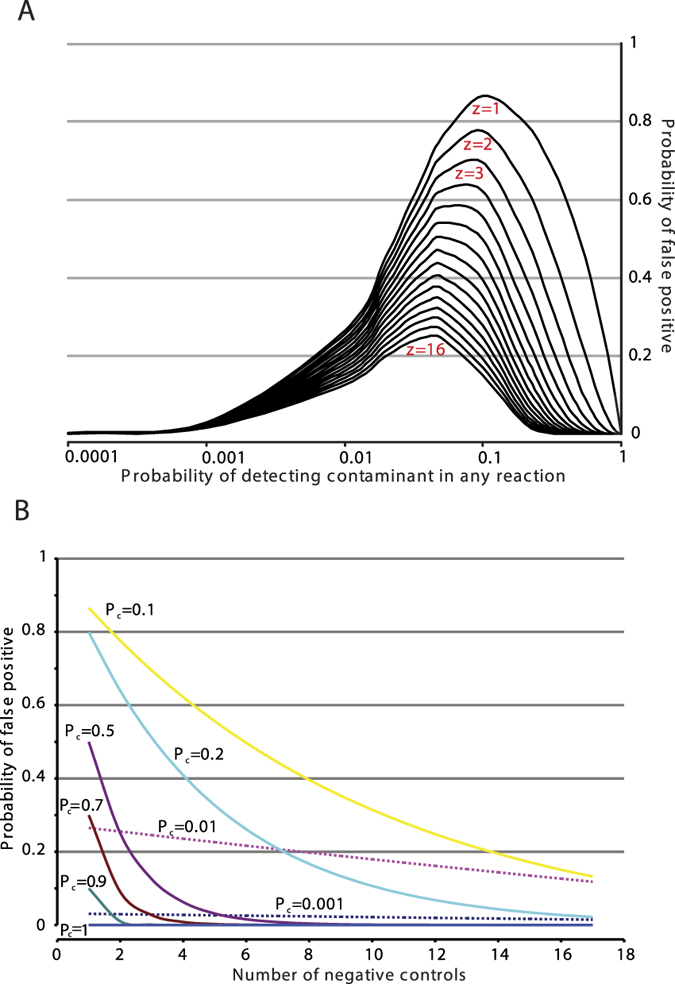
Probability of getting at least one false positive reaction due to low-level contaminants (**A**) as a function of the probability of detecting the contaminant in any reaction and (**B**) as a function of the number of negative controls included in an experiment with 32 reactions.

Based on our observation for low-level contamination we developed a simple mathematical model for low-level contaminants. We assume that the contaminants can be present in any of the reactions of the experiment according to a probabilistic distribution (e.g. Poisson). If the probability of detecting the contaminant in any reaction is *P*_*c*_ ≤ 1 then the probability of getting the false positive scenario (*P*_*f*_) where at least one positive reaction hits a non-negative control reaction and all the negative controls are found negative within an experiment of 32 reactions with *z* negative controls is given by:


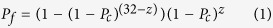


where (1 − *P*_*c*_)^(32−*z*)^ and (1 − *P*_*c*_)^*z*^ are the probabilities that all the non-negative controls and that the negative controls are found negative respectively. It thus follows that 1−(1 − *P*_*c*_)^(32−*z*)^ is the probability that at least one non-negative control is found to be positive. According to this simple mathematical model the probability of getting at least one false positive in a 32-reaction experiment with z negative controls being negative is formally maximized when the probability of detecting the contaminant in any reaction *P*_*c*_ is


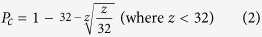


thus if we would use 1 negative control the probability of getting a false positive is maximized when the probability of detecting the contaminant is around 10% and is diminished when the probability approaches 1 or much lower than 0.001 (Fig. 2). We thus categorize the levels of contaminants as ultra low when the probability of detecting them is less than 0.01 and low between 0.01 and 1.

As the model shows the probability of getting a false positive due to an ultra low contaminant (dotted lines, Fig. 2B) decreases linearly by increasing the number of negative controls, and is rapidly diminished for low level contaminants, especially when the probability of low level contaminants is in the area 0.5–1. We, thus, set up the rule that an experiment can be successful if all no-RT controls are found to be negative (i.e. we do not repeat single reactions). Based on this model in our setting by including 1 no-RT control per reaction, the probability of getting false positives for low contaminants is well mitigated. To further eliminate the probability of false positives we also required that the low-copy number (<100 copies/reaction) results need to be replicated within independent experiments. We were able to reasonably control occasional positives in our no-RT controls with the development of well-defined clean room standard operating procedures (see methods).

### Sensitivity and specificity of the qPCR in a model system

We assessed the sensitivity of the assay in two different ways. First, we log10-serial diluted the *in-vitro* transcribed RNA-standards (pre-quantified with RiboGreen) to achieve a final concentration of 1 copy/reaction. We were able to detect the 100 copies/reaction of *in-vitro* transcribed RNA-standard in all replicates. Then, to test for any possible effect of the human plasma in the reaction, we used a pool of 4 HIV plasma samples as buffer to log10-serial-dilute human embryonic carcinoma cell line (NCCIT) supernatant (spiking). NCCITs are shown to produce HK2 particles^8^, thus provide an ideal model system to study the specificity and sensitivity of our assay in both cellular and non-cellular samples. Using the *in-vitro* transcribed RNA standards for the standard curve, we were able to detect and quantify HK2 in the undiluted supernatant and in the plasma-dilutions of it at the level of 1000 copies/ml of plasma in all 3 replicate samples and 100 copies/ml in 2 out of 3 replicate samples (Supplementary Table 1), suggesting that our detection limit can be as low as 184 copies/ml of plasma (although with 67% sensitivity). One copy/reaction is equivalent to 24 copies/ml of plasma. The formula for the calculation of the copy-number equivalents is provided in Supplementary Fig. 1.

The target specificity of the primers in the presence of human gDNA is high as defined by agarose gel electrophoresis (no visual byproducts), melting curve analysis (single peak at 69 °C) and Sanger sequencing of the PCR products. The intra-assay and inter-assay coefficient of variation (n = 10) of the assay was defined as 3.20% and 1.71% respectively.

### Testing non-cellular clinical samples

We finally assessed the RNA levels of HK2 *pol* transcripts in 236 anonymised plasma samples provided by the biobank of the Department of Hygiene, Epidemiology and Medical Statistics, Medical School, University of Athens. Seventy (29.7%) of the samples were positive for HIV-1 RNA (Table 2 - viral loads defined by CE-IVD kits used in the source lab), 70 (29.7%) were positive for HCV RNA and 96 (40.6%) were positive for HBV DNA. We transferred the plasma samples from Greece to the United Kingdom in a cold-chain (dry ice) with in-box temperature monitoring (Supplementary Fig. 2), which confirmed that the temperature did not exceed −50 °C at any time during the transfer.

**Figure 3:**
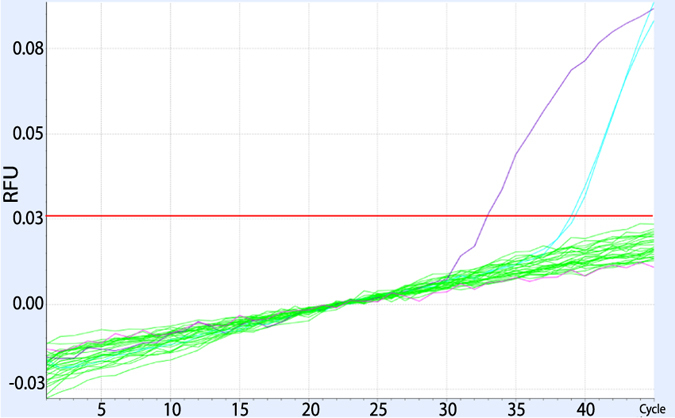
An example of an amplification curve for molecular beacon qPCR for HK2 *pol*. Purple: 10^4^ RNA standard, light blue: 10^2^ RNA standard, green: RT+/RT− samples (N = 14), pink: negative control.

Out of the 236 samples tested in this study, none were found positive for HK2 *pol* transcripts (Fig. 3) even though the detection limit of the assay using log10-dilutions of NCCITs’ supernatant in HIV plasma was as low as 100–1000 copies/ml. In order to test whether the negative result is due to RNA extraction failure, we tested if the RNA extractions were efficient using the reported viral infections of the plasma samples as internal controls. We randomly tested our post DNase-treatment samples by means of a custom in-house SYBR green PCR assay and found them to be positive for HIV or HCV, as reported before their shipment. We further assessed the viral loads of the 14 HIV samples (Table 2, Samples 29–42) using a quantitative SYBR-green assay and we found no significant divergence of the HIV viral loads before and after the shipment (Supplementary Fig. 3A–C). We assessed the total extracted RNA in the same 14 HIV samples using RiboGreen assays and we found it within the expected limits (Supplementary Fig. 4).

Given that the RNA extractions were automated with a highly efficient protocol^35^, we conclude that we find no evidence that the RNA extractions were not successful. The detection limit of our approach using biological material is much lower than the HK2 loads reported in HIV infected individuals^26^,^27^. However, we cannot exclude the possibility that our method fails to detect HK2 virions below the detection limit of 100 copies/ml.

To further explore whether there are HK2 transcripts that could not be detected from our beacon, we tested 14 HIV-1 samples both with our primers alone and with those described by Bhardwaj *et al.*^29^ in SYBR green reactions. We found them negative for HK2 transcripts, while at the same time we were able to detect minimal positive control HK2 RNA (10 copies/reaction) isolated from NCCITs in the same reactions. We were unable to amplify HK2 transcripts either from the same samples or from NCCITs using the primers described by Contreras *et al.*^26^.

## Discussion

In this study, we standardized our laboratory setting for the robust study of HERV expression in clinical samples. We also developed a highly efficient molecular beacon based qPCR assay, which despite being an assay of higher specificity, it has high sensitivity; we were able to detect 100 copies/reaction *in vitro* transcribed RNA standard in all of our experiments and approximately ~7.7 copies/reaction (~184 copies/ml) with 67% sensitivity in our NCCITs supernatant-plasma serial dilutions. This is likely to be due to the optimized DNase treatment used in our experiments; according to our observations, eliminating the total DNase activity in the cDNA preparations increases the efficiency of the detection of lower RNA copy numbers.

The backbone of HERV studies in clinical samples is the use 1:1 no-RT controls in the reactions to be run within the same experiment, reagents and conditions. This setting not only allows monitoring for potential inefficient DNAse treatment, but also serves as a monitor for the inevitable and widespread existence of Human DNA contamination. The later is likely to be low-level and thus much more difficult to take into account as the presence of the contaminant will follow a stochastic distribution. In simple words it will be randomly present in the reactions more or less like the way a digital PCR result. In our pilot experiment this showed up with a pattern of random nonsense positive results. A similar low-level contamination issue was faced with the case of XMRV^36^; however the sources of the contaminants were the reagents or prior use of mouse DNA in the labs of interest^36^. In our case the source of contamination is human DNA, for example a single cell from the person performing the experiment will contaminate the reaction with ~100–200 proviral copies which are present in the human diploid genome. This will be detected at the level of 1000 copy number in the RNA standards as the DNA standard curves amplify earlier (2–3 Ct) than the RNA standards due to the efficiency of reverse transcription. In our setting, a single cell contamination could be interpreted as at least 24,000 copies/ml of plasma.

To control for random contaminants we set the rule that even if only one no-RT reaction would be found positive then the whole experiment should be repeated. Low copy number positives (<100 copies/reaction) should be found positive in at least 2 independent experiments. These two rules completely eliminate the probability of false positives, while the development of standard operating procedures allows for faster, safer and cost-efficient execution of the experiments.

Previous reports have been conflicting whether HK2 RNA can be detected in the plasma of HIV-1 positive patients^29^. We thus investigated the existence of HK2 *pol* transcripts in the plasma of 236 patients infected with HIV-1, HBV or HCV, but we could not detect any HK2 RNA in the samples that we tested. Plasma circulation of HK2 virions could have important health implications - for example for the safety of blood products - although there is no evidence to date that HK2 proviruses can be infectious. On the other hand, two recombinant reconstructed HK2 proviruses have demonstrated low *in vitro* infectivity^24^,^25^. Thus, it is feasible that recombination of up-regulated HK2 RNA could result in the restoration of infectivity^23^.

Given the large pool of samples that we tested, our assay should be able to detect the previously reported HK2 titers (10^4^–10^7^ copies per ml)^26^, expected in 95.33% of HIV positive patients^37^. Given the extensive evaluation of our assay and our approach, we think it is unlikely to be due to the laboratory techniques being different or the sensitivity of the methods used. HERV-K111 and K102 were the strains previously associated with HIV-1 viremia^33^,^34^ and even though they were within the detection spectrum of our assay, we could not detect them in plasma samples. The geographic origin of the patients is an intriguing factor that may have affected the previously reported HK2 viremias, when considering the highly polymorphic profile of HERV-K integrations in the human genome^38^. It is possible one specific HK2 integration (or a population specific polymorphism within HERV-K111 or K102) is crucial for the observed viremias and was present within the previously studied population, but not in ours. One way to test this hypothesis would be by assessing a cohort of HIV-1 (+) samples from a larger geographical area (see geographic origin that was available for some of our HIV-1+ samples in Table 2) in a follow-up study.

A major difference between published reports of HK2 RNA in plasma samples is the DNase treatment. The earlier approaches reported the use of no-RT controls without a prior DNase treatment^26^,^27^,^37^. In follow-up studies, the same researchers reported the use of DNase treatment before RNA extraction either directly on the plasma samples^27^,^28^ or after centrifugation that would result in viral pellets^39^. In our study, we used DNase to remove gDNA contaminants in the post-extraction stage, as two other groups did, the first reporting relatively increased HK2 RNA levels in 16 HIV-positive versus 4 HIV-negative patients^30^, while the second reported no HK2 RNA in the plasma of HIV-1 patients^29^. Performing a DNase treatment before the extraction results in using the DNase enzyme in suboptimal and non-standardized conditions; for example, the activity of DNase in the environment of human plasma could be lower owing to potential inhibitors of DNase activity and other uncontrolled factors in the plasma (e.g. pH or concentration of ions). On the other hand, a study suggested that some HK2 virions might contain DNA instead of RNA, although the majority would still carry RNA^40^; a post RNA-extraction DNase treatment like ours would eliminate these cDNA viral genomes. However, if HK2 virions indeed carry cDNA then a DNase treatment before the nucleic acid extraction should at least sometimes result in positive results in mock-RT controls, which was not previously reported either in the early studies with no DNase treatment^26^,^27^,^37^ or the later reports where they utilized a pre-extraction DNase treatment^27^,^28^.

We showed that the study of HK2 expression *in vivo* is a daunting task, the major risks being gDNA contamination from the environment, at the very least from the person running the experiments. The extensive validation of our approach for contamination-free assessment of HK2 RNA in clinical samples suggests that detectable HK2 transcripts are unlikely to be ubiquitously present in the plasma of patients with HIV-1, HCV or HBV co-infections. We thus suggest that, similarly to ancient DNA experiments, all HERV expression studies (at least for low-transcription activity), should be ran in well-operated clean room facilities with restricted access to a minimal number of users. One-to-one no-RT controls should be ran with the same qPCR protocol and follow all the transcript reactions, while a fully optimized DNase procedure is of paramount importance to salvage low copy number RNA transcripts in the background of massive gDNA contamination.

## Materials and Methods

### Nucleic acid extractions from Human plasma

Fully anonymised plasma samples were provided by the biobank of the Department of Hygiene, Epidemiology and Medical Statistics, Medical School, University of Athens, which routinely acquires written informed consent for the use of the stored samples for research purpose. Sample availability for the described experimental protocols was approved by the Bioethics Committee of the Medical School, University of Athens, and all the methods were carried out in accordance with the approved guidelines.

More specifically, blood samples derived from patients were centrifuged and plasma samples obtained were stored at −70 °C prior to use according to the standard operating procedures of the biobank. We extracted total RNA from 1 ml plasma using the QIAsymphony automated extraction system using the DSP Virus/Pathogen Midi kit (both by Qiagen) eluting in 60 μl. All extractions took place in the Institute of Infection and Global Health at the University of Liverpool and the nucleic acid eluates were transferred to Oxford in a cold-chain.

To test the extraction step, we tested 10 random HCV and 14 random HIV-1 samples with a standard SYBR Green in-house qPCR for the existence of viral cDNA after the DNase and the RT step using the following primers: HCV_F: RC1, HCV_R: RC2^41^ and HIV_F: LTR-OS, HIV_R: LTR-AS^42^. We assessed the extracted total RNA in the 14 HIV samples (Table 2, Samples 29–42) using RiboGreen assays. We further quantified the HIV viral loads in the 14 HIV samples using the same primers (HIV_F: LTR-OS, HIV_R: LTR-AS) in a quantitative approach with standards produced by serial dilutions of a quantified (with PicoGreen) PCR product.

### Cell culture

The NCCIT cells are cultured in RPMI growth medium supplemented with 10% v/v foetal bovine serum, 1% v/v penicillin-streptomycin and 2 mM L-glutamine. They are allowed to grow to 100% confluence in a T75 flask (Falcon, VWR UK). The cells were typically sub-cultured twice a week using trypsin-EDTA to dislodge them from the substratum. The cells were used to seed a 12 well cluster plate at 5 × 10^5^ cells/well and the next day they were treated with IL-1β (100 pg/mL). The cells were harvested 24 h post-treatment by centrifugation and the cell pellets were stored at −80 °C prior to RNA extraction. Total RNA was extracted using the Qiagen QIAshredder (79656) and the Qiagen RNeasy Mini kit (74106) according to the manufacturer’s protocol.

### Clean rooms and standard operating procedures (SOPs)

All the subsequent pre-PCR steps were performed in a dedicated clean lab where amplified products (post-PCR) are not permitted. Access is restricted to a limited number of trained personnel to keep genomic DNA contamination of the environment to minimum levels. Briefly, the clean lab is physically split in two self-contained clean rooms and a storage space. The first clean room is restricted to not allow handling of any nucleic acid, while the second clean room allows for handling of (non-amplified) nucleic acids. The nucleic acid eluates were stored at −80 °C in the storage space of the clean lab. The workflow is “one-way” from the first to the second clean room and then to the other lab space were PCR and post-PCR handling take place. The preparation of the master mixes in both of these clean rooms is performed in designated hoods after rigorous UV decontamination and the circulating air is cleaned through HEPA filtration.

### Removal of genomic DNA, negative controls and optimization of RT

We DNase-treated the nucleic acid eluates using a modified protocol of TURBO DNA-free Kit (AM1907, Invitrogen). Briefly, we used 2 μl of DNase in 2.4 μl of the reaction buffer per 20 μl of RNA. After 30 min incubation at 37 **°**C, we inactivated the DNase and removed divalent cations using the inactivation reagent supplied in the kit. The latter was removed by centrifugation at 10,000 g for 1.5 min and the supernatant was heated at 70 **°**C for 10 min to eliminate any remnant DNase activity. For each RNA sample, we prepared an RT (+) and an RT (−) mock reaction (no enzyme added) using the reverse primer of each downstream qPCR assay and the SuperScript III Reverse Transcriptase (18080, Life Technologies). The use of the reverse primer instead of random hexamers further increased the sensitivity of the RT reactions, as we observed a 4-cycle shift of the cycle thresholds (Ct) when testing the 10^4^ and 10^3^ RNA standards. We were also able to consistently amplify the 10^2^ RNA standard only by the use of the reverse primer.

### Quantitative PCR

We designed the qPCR primers HK2_5b_For: 5′-AAT TGA CTG TTA YAC ATT TCT RC-3′, HK2_3_Rev: 5′-CCG AAT CCA ATT AAT ATC TCC-3′ and the beacon probe [6FAM]-CCG AGC CCA TCT GAT AAG ATC CAA ACC TCT ACT CGG CTC GG-[BHQ1]. Our assay targets the reverse transcriptase and more specifically the catalytic motif (YMDD) as this is biologically relevant and, thus, more conserved among infectious retroviruses.

**Figure 4:**
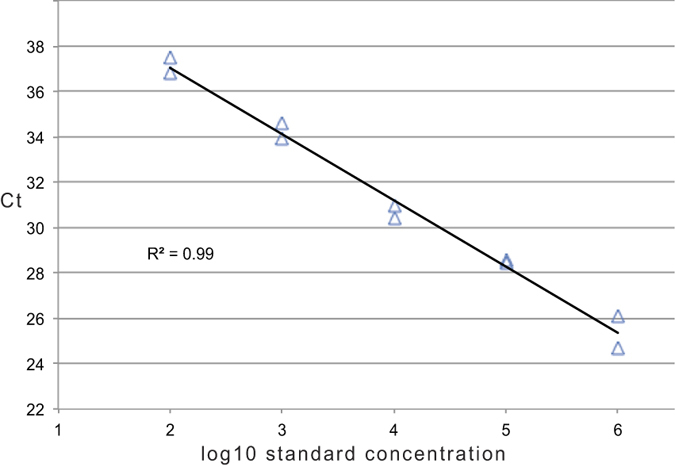
RNA Standard Curve. Log10 serial dilutions of DNase-treated 10^8^ RNA standard, tested in duplicate. Threshold cycle (Ct) values are plotted against the number of RNA copies/reaction.

We constructed the RNA standards using MAXIscript T7 Transcription Kit (AM1312, Invitrogen) and the HK2 *pol* DNA template of K113 that was obtained from Invitrogen. We DNase-treated the RNA transcripts and, after visualizing them in RNA gels (Formaldehyde-Free RNA Gel Kit, N726-KIT, Amresco/VWR chemicals) to confirm their integrity, we quantified them using Quant-iT Ribo-Green (R11409, Invitrogen). We constructed the RNA standard curve by log10 serial dilutions of RNA standard (kit-included, pre-aliquoted) to achieve final control concentration 10^6^ down to 10^2^ copies/reaction (Fig. 4).

To exclude the possibility of missing expression of proviruses due to the increased specificity of the molecular beacon probe, we tested 14 random HIV samples (Table 2, samples 29–42) using both our and also previously described HK2 pol primers^26^,^29^ in combination with Platinum SYBR Green (11744, Invitrogen), using NCCIT cDNA as a positive control.

Using multiple melt curves, we further examined the molecular beacon specificity against all the possible variations of our target sequence found in a previously described alignment of all (full-length or almost full-length) HERV-K proviruses^6^.

### Ethics approval and consent to participate

Sample availability was approved by the Bioethics Committee of the Medical School, University of Athens.

## Additional Information

**How to cite this article**: Karamitros, T. *et al.* A contaminant-free assessment of Endogenous Retroviral RNA in human plasma. *Sci. Rep.* **6**, 33598; doi: 10.1038/srep33598 (2016).

## Supplementary Material

Supplementary Information

## Figures and Tables

**Table 1:** Detectable and not detectable HERV-K HML-2 proviruses based on multiple melt curve analysis (adapted from Subramanian *et al.* 6).

Locus	Coordinates (hg19) Chr:Start-End	Name	Orient.	Estim. Age	ORFs	LTR Type	Detected
1p31.1	1:75842771–75849143	K4, K116, ERVK-1	(+)	<2	gag	Hs	−
1p34.3	1:36955490–36956728		(−)	N/A		Hs	−
1p36.21a	1:12840260–12846364		(−)	N/A	gag	5B	+
1p36.21b	1:13458305–13467826	K(OLDAL023753), K6, K76	(+)	22.47–40.68	gag	5B	+
1p36.21c	1:13678850–13688242	K6, K76	(+)	22.69–41.09	gag	5B	+
1q21.3	1:150605284–150608361		(−)	N/A		Hs	−
1q22	1:155596457–155605636	K102, K(C1b), K50a, ERVK-7	(−)	<2		Hs	+
1q23.3	1:160660575–160669806	K110, K18, K(C1a), ERVK-18	(+)	7.81–14.14		Hs	+
1q24.1	1:166574603–166580258	K12	(−)	14.17–25.65		5B	−
1q32.2	1:207808457–207812636		(−)	N/A		Hs	−
1q43	1:238925595–238927773		(−)	N/A		5B	−
2q21.1	2:130719538–130722209		(−)	N/A		Hs	−
3p12.3	3:75600465–75609150		(+)	N/A		5A	−
3p25.3	3:9889346–9896236	K11, ERVK-2	(−)	12.13–21.96*		Hs	−
3q12.3	3:101410737–101419859	K(II), ERVK-5	(+)	5.51–9.98		Hs	+
3q13.2	3:112743479–112752282	K106, K(C3), K68, ERVK-3	(−)	<2	gag	Hs	+
3q21.2	3:125609302–125618416	K(I), ERVK-4	(+)	4.8–8.69		Hs	+
3q24	3:148281477–148285396	ERVK-13	(−)	N/A	gag	Hs	+
3q27.2	3:185280336–185289515	K50b, K117, ERVK-11	(−)	<2	gag, pol	Hs	+
4p16.1a	4:9123515–9133075	K17b	(+)	16.84–30.49		5A	−
4p16.1b	4:9659588–9668650	K50c	(+)	17.19–31.13		5A	−
4p16.3a	4:234989–239459		(+)	N/A		5B	+
4p16.3b	4:3980069–3988631	K77	(−)	11.1–20.1		5A	−
4q13.2	4:69463709–69469223		(+)	17.09–30.95	env	5A	−
4q32.1	4:161579938–161582360		(+)	N/A		Hs	−
4q32.3	4:165916840–165924068	K5, ERVK-12	(+)	9.24–16.73		Hs	−
4q35.2	4:191027414–191034701		(−)	13.07–23.67		5A	−
5p12	5:46000159–46010002		(−)	13.39–24.24		Hs	+
5p13.3	5:30487114–30496205	K104, K50d	(−)	6.32–11.44		Hs	+
5q33.2	5:154016502–154024214	K18b	(−)	13.03–23.6	env	5A	−
5q33.3	5:156084717–156093896	K107/K10,	(−)	<2	gag, pol	Hs	+
6p11.2	6:57623896–57628704	K23	(+)	9.38–16.99		5A	−
6p21.1	6:42861409–42871367	K(OLDAL035587),	(−)	9.83–17.81		5B	+
6p22.1	6:28650367–28660735	K(OLDAL121932),	(+)	19.55–35.4		Hs	−
6q14.1	6:78427019–78436083	K109, K(C6),	(−)	<2	gag, env	Hs	+
6q25.1	6:151180749–151183574		(+)	N/A	env	5B	−
7p22.1a	7:4622057–4631528	K108L, K	(−)	<2	pol, env	Hs	+
7p22.1b	7:4630561–4640031	K108R, ERVK-6	(−)	<2	pol, env	Hs	+
7q11.21	7:65469689–65472384		(−)	N/A		5B	−
7q22.2	7:104388369–104393266	ERVK-14	(−)	N/A	gag	Hs	+
7q34	7:141450926–141455903	K(OLDAC004979),	(−)	N/A	gag	Hs	+
8p22	8:17765202–17773940		(−)	N/A		HML11	−
8p23.1a	8:7355397–7364859	K115, ERVK-8	(−)	4.87–8.82*	gag, pol, env	Hs	+
8p23.1b	8:8054700–8055725	K27	(+)	16.09–29.13		5A	−
8p23.1c	8:12073970–12083497		(−)	15–27.17		5A	−
8p23.1d	8:12316492–12326007	KOLD130352	(−)	15.22–27.56		5A	−
8q11.1	8:47175650–47183661	K70, K43	(−)	23.46–42.48		5A	−
8q24.3a	8:140472149–140475236		(−)	N/A		Hs	−
8q24.3b	8:146246648–146254211	K29	(−)	12.33–22.33	env	5A	−
9q34.11	9:131612515–131619736	K31	(+)	12.43–22.51		5A	+
9q34.3	9:139674766–139684228	K30	(−)	15.46–28		5B	−
10p12.1	10:27182399–27183380	K103, K(C10)	(+)	1.61–2.91	gag, pol	Hs	+
10p14	10:6867109–6874635	K(C11a), K33,	(−)	7.36–13.32		Hs	+
10q24.2	10:101580569–101587716	ERVK–17, c10_B	(−)	N/A	gag	Hs	+
11p15.4	11:3468656–3478209	K7	(−)	15.44–27.95		5A	+
11q12.1	11:58767448–58773196		(+)	N/A	env	5B	−
11q12.3	11:62135963–62150563	K(OLDAC004127)	(−)	19.46–35.24*	pol	Hs	+
11q22.1	11:101565794–101575259	K(C11c), K36,	(+)	<2	pol	Hs	+
11q23.3	11:118591724–118600883	K(C11b), K37,	(−)	13.35–24.18	gag	Hs	+
12p11.1	12:34772555–34782217	K50e	(−)	39.23–71.02		5A	−
12q13.2	12:55727215–55728183		(+)	<2	gag, pol	Hs	+
12q14.1	12:58721242–58730698	K(C12), K41,	(−)	<2	gag, pol, env	Hs	+
12q24.11	12:111007843–111009325		(+)	N/A		Hs	−
12q24.33	12:133667120–133673132	K42	(−)	7.07–12.81		5A	−
14q11.2	14:24480625–24484121	K(OLDAL136419),	(−)	11.98–21.69	gag	5A	−
14q32.33	14:106139659–106142540		(+)	N/A		5A	−
15q25.2	15:84829020–84832364		(+)	N/A		5B	−
16p11.2	16:34231474–34234142		(+)	N/A		Hs	−
16p13.3	16:2976160–2977661	K(OLDAC004034	(+)	N/A		5B	−
17p13.1	17:7960357–7967219		(+)	N/A		HML11	−
19p12a	19:20387400–20397512	K52	(+)	29.71–53.79*		5B	+
19p12b	19:21841536–21841542	K113	(−)	<2	gag, pol, env	Hs	+
19p12c	19:22757824–22764561	K51	(+)	12.96–23.47*		Hs	−
19p13.3	19:385095–387637	ERVK-22	(+)	N/A		Hs	−
19q11	19:28128498–28137361	K(C19), ERVK-	(−)	N/A	gag, env	Hs	+
19q13.12a	19:36063207–36067434		(−)	N/A		Hs	−
19q13.12b	19:37597549–37607066	K(OLDAC012309),	(−)	22.87–41.42*		Hs	+
19q13.41	19:53248274–53252591		(−)	N/A	pol	5B	+
19q13.42	19:53862348–53868044	LTR13	(+)	N/A	env	5B	−
20q11.22	20:32714750–32724384	K(OLDAL136419),	(+)	15.74–28.5		5B	−
21q21.1	21:19933916–19941962	K60, ERVK-23	(−)	3.46–6.27		Hs	+
22q11.21	22:18926187–18935307	K101, K(C22),	(+)	1.84–3.34		Hs	+
22q11.23	22:23879930–23888810	K(OLDAP000345),	(+)	21.64–39.18*	gag	5B	+
Xq11.1	X:61959549–61962054		(+)	N/A	env	5B	−
Xq12	X:65684132–65686184		(−)	N/A		5A	−
Xq28a	X:153817163–153819562	K63	(+)	N/A	gag	5B	−
Xq28b	X:153836675–153844015	K63	(−)	14.65–26.52	gag	5B	−
Yp11.2	Y:6826441–6833384		(−)	N/A		5A	−
Yq11.23a	Y:26397837–26401035		(−)	N/A		5B	−
Yq11.23b	Y:27561402–27564601		(+)	N/A		5B	−

**Table 2:** Demographic and clinical characteristics of HIV-1 infected patients (n.a. – not available).

Sample	Subtype	Risk group	Gender	Ethnicity	RNA Quantitative*
1	n.a.	n.a.	F	n.a.	2.88E + 05
2	CRF14	IDU	M	SOUTH & SOUTH EAST ASIA	4.63E + 01
3	n.a.	MSM	M	n.a.	3.83E + 02
4	n.a.	MSM	M	n.a.	1.26E + 03
5	B	MSM	M	W. EUROPE	8.27E + 02
6	B	MSM	M	HELLENIC	2.24E + 04
7	n.a.	MSM	M	HELLENIC	8.88E + 03
8	n.a.	MSM	M	n.a.	1.37E + 04
9	n.a.	MSM	M	HELLENIC	3.27E + 02
10	n.a.	MSM	M	n.a.	1.46E + 04
11	CRF14	IDU	M	HELLENIC	1.27E + 03
12	n.a.	HETERO	M	HELLENIC	1.23E + 02
13	n.a.	HETERO	M	n.a.	8.47E + 01
14	n.a.	n.a.	M	n.a.	2.68E + 03
15	B	IDU	M	HELLENIC	4.57E + 01
16	n.a.	n.a.	F	n.a.	1.97E + 05
17	n.a.	n.a.	M	n.a.	2.16E + 03
18	n.a.	IDU	M	n.a.	1.65E + 03
19	n.a.	MSM	M	HELLENIC	4.47E + 04
20	n.a.	MSM	M	n.a.	3.65E + 04
21	n.a.	MSM	M	HELLENIC	8.02E + 01
22	n.a.	MSM	M	n.a.	2.30E + 02
23	n.a.	MSM	M	n.a.	8.96E + 04
24	A1	MSM	M	HELLENIC	1.29E + 02
25	n.a.	n.a.	M	n.a.	1.20E + 05
26	n.a.	n.a.	M	n.a.	4.40E + 01
27	CRF14/CRF35	IDU	M	N.AFRICA-MIDDLE EAST	6.04E + 04
28	n.a.	n.a.	F	n.a.	2.27E + 02
29	n.a.	IDU	M	n.a.	5.48E + 01
30	A1	MSM	M	n.a.	3.23E + 03
31	n.a.	MSM	M	HELLENIC	1.78E + 02
32	n.a.	MSM	M	n.a.	2.70E + 02
33	n.a.	n.a.	M	n.a.	4.33E + 04
34	A1	MSM	M	HELLENIC	3.06E + 05
35	n.a.	MSM	M	n.a.	3.70E + 04
36	n.a.	MSM	M	n.a.	1.08E + 04
37	n.a.	MSM	M	HELLENIC	3.55E + 04
38	n.a.	MSM	M	n.a.	2.96E + 03
39	n.a.	HETERO	F	E. EUROPE	1.34E + 04
40	n.a.	IDU	F	n.a.	4.49E + 04
41	B	HETERO	M	n.a.	1.84E + 02
42	n.a.	MSM	M	n.a.	3.38E + 04
43	CRF14	IDU	M	HELLENIC	3.07E + 03
44	n.a.	MSM	M	n.a.	5.13E + 01
45	n.a.	MSM	M	n.a.	9.66E + 03
46	n.a.	n.a.	M	n.a.	2.18E + 05
47	A1	IDU/MSM	M	HELLENIC	5.21E + 04
48	CRF14/B	IDU	M	SOUTH EAST ASIA	2.58E + 02
49	B	HETERO	F	HELLENIC	7.31E + 04
50	n.a.	MSM	M	n.a.	6.66E + 01
51	C	HETERO	M	n.a.	5.79E + 01
52	n.a.	HETERO	F	n.a.	1.12E + 03
53	B	IDU	M	HELLENIC	6.07E + 03
54	n.a.	HETERO	M	C. EUROPE	2.15E + 04
55	n.a.	n.a.	M	n.a.	3.25E + 04
56	n.a.	MSM	M	n.a.	5.01E + 01
57	B	IDU	M	HELLENIC	1.12E + 03
58	n.a.	HETERO	F	n.a.	6.29E + 03
59	n.a.	MSM	M	n.a.	1.05E + 02
60	n.a.	MSM	M	n.a.	9.29E + 01
61	CRF35/CRF14	IDU	F	HELLENIC	1.65E + 05
62	n.a.	HETERO	M	n.a.	3.43E + 02
63	CRF14	IDU	M	HELLENIC	1.73E + 02
64	n.a.	n.a.	M	n.a.	3.47E + 01
65	n.a.	HETERO	F	n.a.	2.06E + 03
66	CRF14	IDU	M	HELLENIC	1.54E + 02
67	n.a.	MSM	M	n.a.	3.92E + 04
68	n.a.	MSM	M	n.a.	3.25E + 04
69	n.a.	n.a.	M	n.a.	2.97E + 02
70	A1	MSM	M	HELLENIC	4.46E + 01

^*^Viral loads in copies/ml of plasma defined by CE-IVD kits in the source lab (pre-shipment).
