# Supplementary material for: A contaminant-free assessment of Endogenous Retroviral RNA in human plasma
Source: Sci Rep. 2016 Sep 19;6:33598. doi: 10.1038/srep33598 (PMC5027517; doi:10.1038/srep33598)

Supplementary Material

A contaminant-free assessment of Endogenous Retroviral RNA in human plasma

Timokratis Karamitros[1], Dimitrios Paraskevis[2], Angelos Hatzakis[2], Mina Psichogiou[3], Ioannis Elefsiniotis[4], Tara Hurst[1], Anna-Maria Geretti[5], Apostolos Beloukas[5], John Frater[6], Paul Klenerman[6], Aris Katzourakis[1], Gkikas Magiorkinis[1#]

1.Department of Zoology, University of Oxford

2.Department of Hygiene, Epidemiology and Medical Statistics, Medical School, University of Athens

3.First Department of Internal Medicine, Medical School, University of Athens

4.Academic Department of Internal Medicine-Hepatogastroenterology, General and Oncology Hospital 'Agioi Anargyroi'

5.Institute of Infection and Global Health, University of Liverpool, 6.Nuffield Department of Medicine, University of Oxford

#Corresponding Author: Gkikas Magiorkinis: gkikas.magiorkinis@zoo.ox.ac.uk

e-mail addresses: timokratis.karamitros@zoo.ox.ac.uk, dparask@med.uoa.gr, ahatzak@med.uoa.gr, mpsichog@yahoo.gr, ielefs@nurs.uoa.gr tara.hurst@zoo.ox.ac.uk, A.M.Geretti@liverpool.ac.uk, A.Beloukas@liverpool.ac.uk, john.frater@ndm.ox.ac.uk, paul.klenerman@medawar.ox.ac.uk, aris.katzourakis@zoo.ox.ac.uk, gkikas.magiorkinis@zoo.ox.ac.uk

Suppl. Table 1: Sensitivity of the assay in biological material. Absolute quantification of HK2 pol in undiluted and in log10-serial-diluted NCCIT cell-supernatant in HIV plasma (spiking).

Type	Cq	Amplification	Estimated copies/reaction	Average copies/reaction	Estimated* copies/ml	Expected** copies/ml
NCCIT SUPERNATANT	30.86	TRUE	4397.14	4236.33	101672	
NCCIT SUPERNATANT	31.92	TRUE	2073.52			
NCCIT SUPERNATANT	30.37	TRUE	6238.33			
SUPERNAT plasmaSpike 1/10	35.10	TRUE	216.39	346.11	8307	10167
SUPERNAT plasmaSpike 1/10	34.10	TRUE	440.31			
SUPERNAT plasmaSpike 1/10	34.30	TRUE	381.62			
SUPERNAT plasmaSpike 1/100	37.19	TRUE	48.97	27.63	**663**	**1017**
SUPERNAT plasmaSpike 1/100	39.11	TRUE	12.51			
SUPERNAT plasmaSpike 1/100	38.35	TRUE	21.41			
SUPERNAT plasmaSpike 1/1000	39.64	TRUE	8.57	7.67	184	102
SUPERNAT plasmaSpike 1/1000	39.97	TRUE	6.77			
SUPERNAT plasmaSpike 1/1000		FALSE				
Standard, 10000 copies/reaction	30.23	TRUE	6863.48	10590.70		
Standard, 10000 copies/reaction	29.20	TRUE	14317.92			
Standard, 1000 copies/reaction	32.63	TRUE	1251.27	1039.42		
Standard, 1000 copies/reaction	33.21	TRUE	827.56			
Standard, 100 copies/reaction	36.61	TRUE	73.71	103.52		
Standard, 100 copies/reaction	35.78	TRUE	133.33			
Negative		FALSE				

* Rounded, based on the formula presented in Suppl. Figure 1.
**Expected copies after plasma spiking

Suppl. Figure 1: Calculation of equivalence between copies/reaction and copies/ml plasma

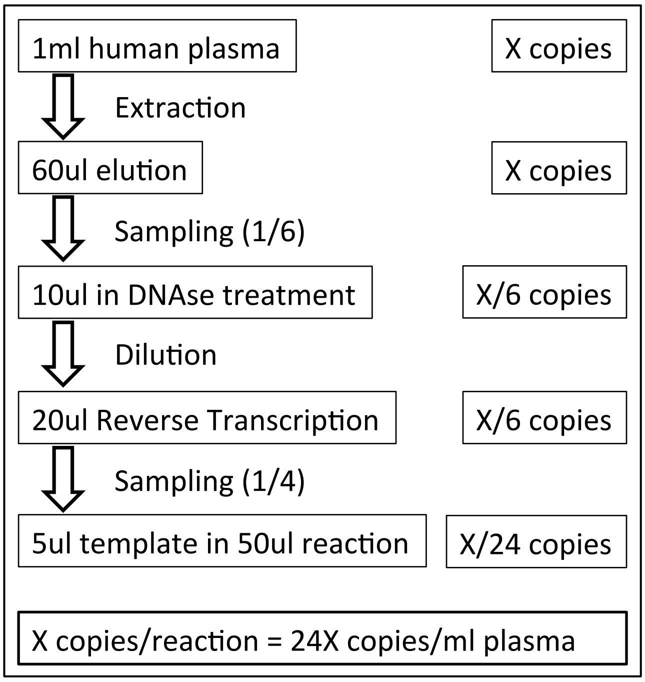

Suppl. Figure 2: Temperature log during the transport of the plasma samples from Greece to the United Kingdom. Dry ice was replenished during the shipment.

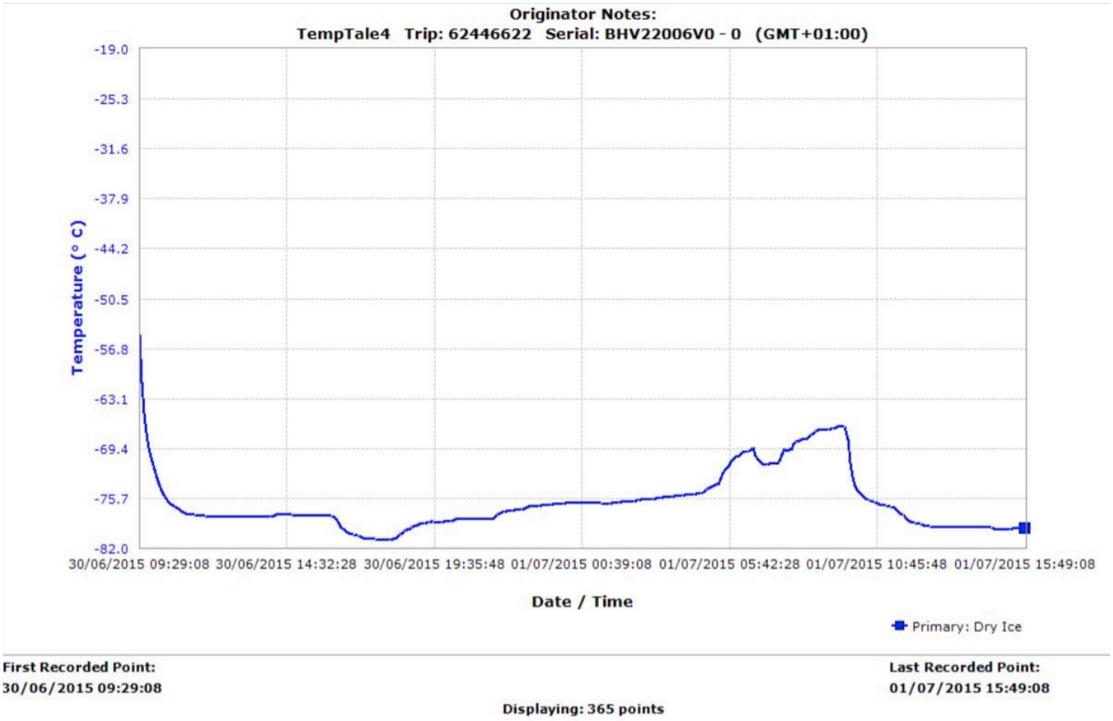

Suppl. Figure 3: SYBR-Green quantification of 14 HIV samples and comparison with pre-shipment measurements. Standards were built after absolute quantification of PCR amplicon with PicoGreen.

A) Standard Curve (E=1.922, r^2=0.9961)

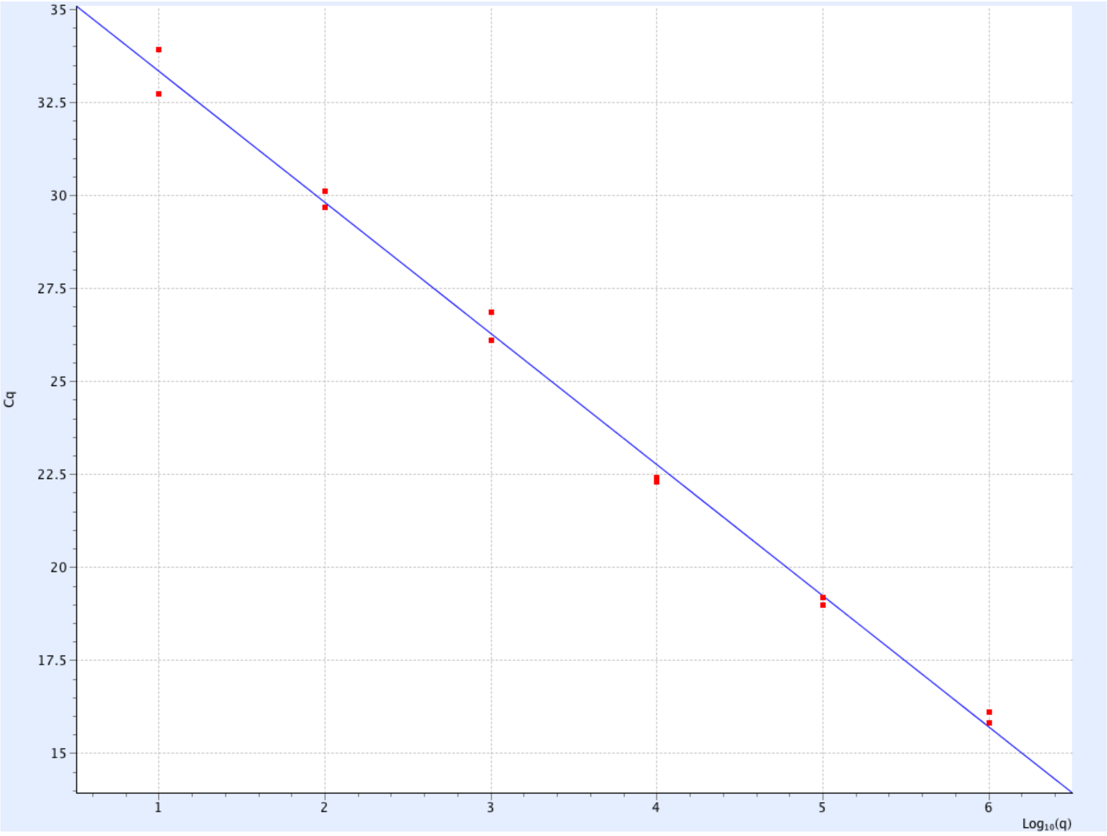

B) Pre- / post- shipment quantification

Sample	SYBR-green copies/reaction	Estimated* copies/ml	pre-shipment copies/ml
HIV29	2.55E+00	6.13E+01	5.48E+01
HIV30	1.47E+02	3.52E+03	3.23E+03
HIV31	6.45E+00	1.55E+02	1.78E+02
HIV32	8.04E+00	1.93E+02	2.70E+02
HIV33	9.73E+02	2.34E+04	4.33E+04
HIV34	3.81E+04	9.14E+05	3.06E+05
HIV35	1.42E+03	3.41E+04	3.70E+04
HIV36	3.19E+02	7.66E+03	1.08E+04
HIV37	8.91E+02	2.14E+04	3.55E+04
HIV38	1.06E+02	2.54E+03	2.96E+03
HIV39	4.71E+02	1.13E+04	1.34E+04
HIV40	1.23E+03	2.95E+04	4.49E+04
HIV41	7.65E+00	1.84E+02	1.84E+02
HIV42	6.05E+02	1.45E+04	3.38E+04

* Based on the formula presented in Suppl. Figure 1.

C) Regression line of pre- vs. post- shipment HIV viral loads (log10-transformed values). The 95% confidence intervals of the intercept and the slope were -0.5008 to 0.3388 and 0.8945 to 1.110 respectively.

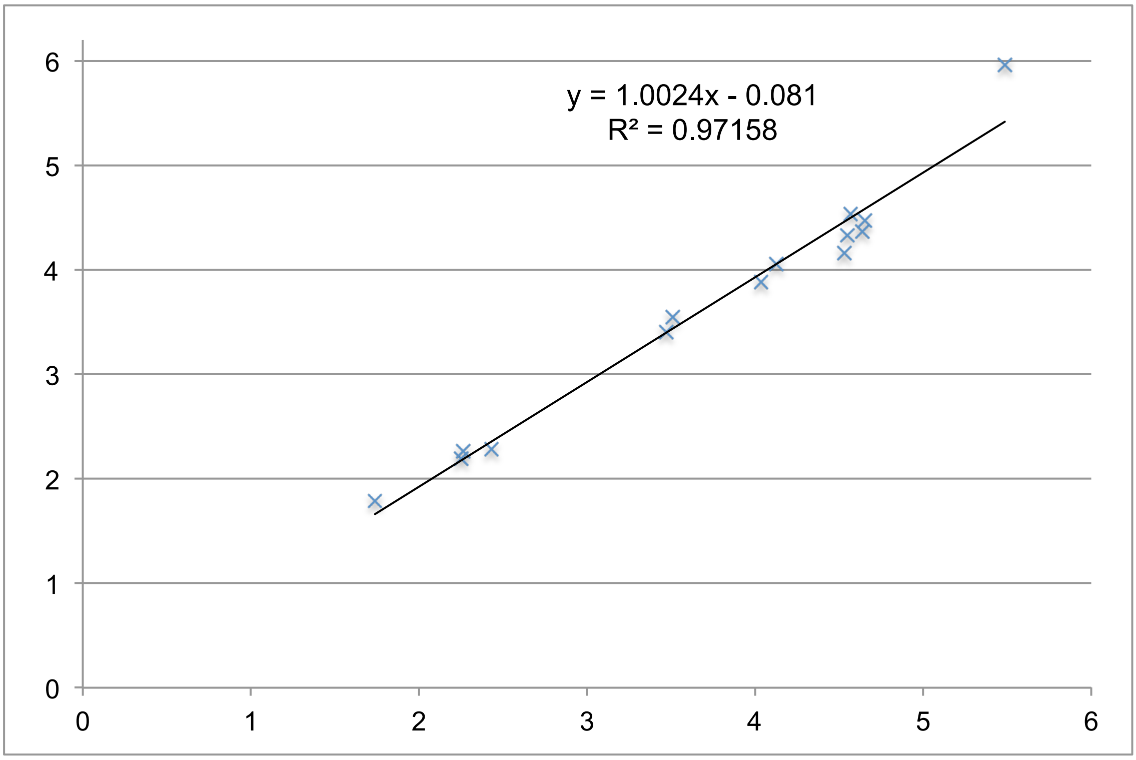

Suppl. Figure 4: Absolute quantification total RNA with Ribogreen. RNA extracted from 14 HIV samples (1 ml plasma) was DNAse treated and quantified using the kit-included rRNA standards.

Standard Curve

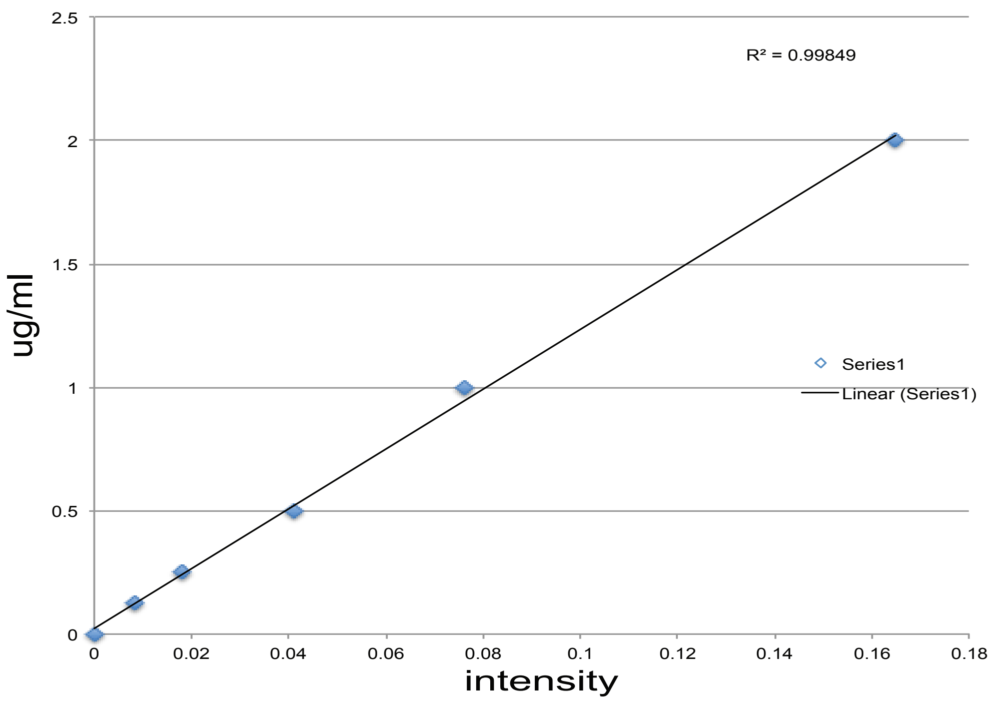

Total RNA in 14-HIV samples

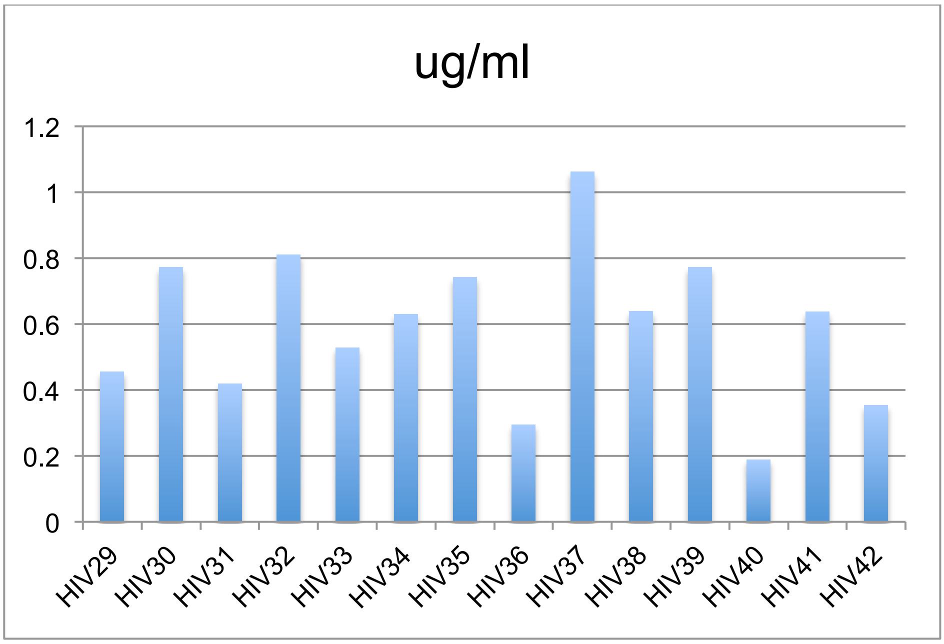

Supplement: Supplementary Information [file srep33598-s1.pdf]
